# Trends in the Retinal Nerve Fiber Layer Thickness Changes with Different Degrees of Visual Field Defects

**DOI:** 10.1155/2020/4874876

**Published:** 2020-04-12

**Authors:** Wenhui Geng, Dabo Wang, Jing Han

**Affiliations:** Affiliated Hospital of Qingdao University, Qingdao, China

## Abstract

**Purpose:**

To explore the disease progression of primary open-angle glaucoma (POAG) in individuals with different degrees of VF defects by analyzing the trends in retinal nerve fiber layer (RNFL) changes at each stage.

**Methods:**

A total of 39 patients (77 eyes) were divided into three groups based on the severity of glaucomatous visual field (VF) loss: the first group included patients with mild baseline VF defects (mild group; *n* = 21 eyes). The second group included patients with moderate VF defects (moderate group; *n* = 18 eyes). The third group included patients with severe baseline VF defects (severe group; *n* = 38 eyes). For all patients, slit-lamp biomicroscopy of the anterior and posterior segments and detailed fundus and optic disc inspections were performed, the intraocular pressure (IOP) was measured by Goldman tonometry, best-corrected visual acuity (BCVA) was measured, the RNFL thickness was measured by OCT, and the VF was assessed by the Octopus perimeter. All the groups were followed up postoperatively for 18 months.

**Results:**

The mean RNFL thickness was recorded for all the visits. Using simple linear regression analysis, we found that the *R*^2^ values of the three groups were 0.988, 0.982, and 0.814, respectively, and the slopes of mean RNFL thickness changes for mild, moderate, and severe baseline VF defects were −0.088, −0.082, and −0.015, respectively. Moreover, we used simple linear regression analysis to explore whether and how the speed of RNFL thinning differs across groups. The *R*^2^ values of the three groups were 0.982, 0.978, and 0.805, respectively, and the slopes for mild, moderate, and severe baseline VF defects were 0.089, 0.085, and 0.017, respectively.

**Conclusion:**

The rate of RNFL thinning is linear; RNFL thinning is the fastest in individuals with mild baseline VF defects, followed by those with moderate baseline VF defects. In individuals with severe VF defects, changes in the RNFL thickness do not appropriately reflect the progression of the disease. The clinical trial is registered with ChiCTR2000028975.

## 1. Introduction

Primary open-angle glaucoma (POAG) is a common clinical eye disease that is irreversible and causes blindness; it is a type of optic neuropathy characterized by the loss of retinal ganglion cells and their axons [1]. An estimated 10% to 39% of patients with POAG present with advanced glaucoma, and many are asymptomatic at the time of diagnosis [2–4]. Therefore, it is important to detect the occurrence and development of POAG early. Clinicians need to determine whether a condition is progressing, whether the current therapy is suitable for a patient, and whether the therapy needs to be intensified. At present, the visual field (VF) examination is still considered the gold standard for the diagnosis of glaucoma [5], while in view of the differences in the education level and psychological quality of patients, the false-positive rate of VF examination results is high, the repeatability is poor, and it is easy to cause misjudgment [6]. And it is widely recognized that glaucoma is a multifactorial optic neuropathy that is characterized by continuous processes, including progressive neurodegeneration of retinal ganglion cells (RGCs) and their axons, resulting in retinal nerve fiber layer (RNFL) attenuation, damage to the optic nerve head, and irreversible VF loss [7]. Since the thickness of the RNFL can effectively reflect the number of axons in nerve cells, we can estimate retinal ganglion cell survival by determining the thickness of the RNFL using stratus optical coherence tomography (OCT) [8].

Previous studies have shown that the VF defects progress linearly with the progression of the disease, and the linear relationship differs according to the baseline VF defects. The mildest defects are associated with the fastest progression, moderate defects are associated with the second fastest progression, and severe defects are associated with the slowest progression [9]. In clinical practice, to determine the progression of the disease in a timely manner, patients usually need regular vision and OCT examinations every 3 to 6 months. While as it has been mentioned above, the speed of VF deterioration of patients with different VF defects differs. Therefore, it is unclear whether maintaining the original examination frequency is suitable for patients with different baseline VF defects for that inappropriate inspection frequency might lead to a failure to detect the progression of the disease. Although RNFL thickness parameters have high sensitivity in identifying glaucoma, there may be less agreement between perimeters and nerve fiber layer instruments in classifying eyes with glaucoma. Thus, different techniques may identify different characteristics of glaucomatous damage [10]. To explore the disease progression in patients with different degrees of VF defects, we need to study the trends in RNFL changes that occur with different severities of VF defects.

In this study, RNFL thickness was measured by OCT, the Octopus perimeter was used to perform the VF examination, and the patients were observed and followed up for 18 months. The trends in the RNFL thickness changes in patients with POAG and different degrees of VF defects were explored so that reasonable follow-up frequencies for POAG patients can be determined and disease progression can be detected early.

## 2. Methods

This prospective study was conducted in the Department of Ophthalmology at the Affiliated Hospital of Qingdao University between September 2017 and October 2019. In the current study, patients with POAG in the outpatient clinic were observed and followed up. This study adhered to the tenets of the Declaration of Helsinki and was approved by the Ethics Committee of the Affiliated Hospital of Qingdao University. Informed consent was obtained from each patient for enrollment in the study. The participants were divided into three groups based on the severity of glaucomatous VF loss: the first group included patients with mild baseline VF defects (mild group; *n* = 21 eyes). The second group included patients with moderate VF defects (moderate group; *n* = 18 eyes). The third group included patients with severe baseline VF defects (severe group; *n* = 38 eyes).

This was a prospective cohort study. Outpatients with POAG were selected from September 2017 to April 2018 and followed up for eighteen months from September 2017 to October 2019. The inclusion criteria consisted of a POAG diagnosis [11], no history of surgery within 3 months before the start of the examination, best-corrected visual acuity of 20/40 or better, the intraocular pressure may fluctuate, but peak IOP does not exceed 21 mmHg, corneal thickness of all subjects was within the normal range (500 *μ*m∼550 *μ*m), using regular IOP-lowering drugs to control IOP at a stable level within 6 months before the start of follow-up, no other eye diseases or serious systemic diseases that can affect the reliability of the results, availability to participate in the study, and reliable data. The exclusion criteria consisted of poor intraocular pressure control (<10 mmHg or ≥21 mmHg) for more than 3 consecutive measurements in a single day and serious diseases found during the follow-up period, and pregnant women and nursing mothers were also excluded.

At baseline, patients diagnosed with POAG were selected and completed the ophthalmic examination, which included a review of their medical history, slit-lamp biomicroscopy of the anterior and posterior segments, and detailed fundus and optic disc inspections. IOP was measured by Goldman tonometry; this examination and the best-corrected visual acuity, fundus, VF, and RNFL examinations were conducted to identify various eye diseases that affect the examination accuracy and exclude patients with these diseases. All participants were observed and followed up once every 3 months for 18 months.

The IOP was measured three times continuously by the Goldman tonometer, and the average value was selected as the current intraocular pressure. If the IOP of an eye of a patient was measured 3 times on the same day to be > 21 mmHg, the eye was excluded from the analysis.

After using 0.5 g/L compound tropicamide to fully dilute the pupil, centered on the optic papilla, with a diameter of 3.4 mm, the average thickness of the four quadrants (upper, lower, nasal, and temporal) of the circular RNFL section around the disc was measured by Heidelberg optical coherence tomography. The imaging quality of the OCT scans ≥45, the segmentation was not adjusted, and the thickness of OCT scans is 1.9 mm.

Using the G2 program for the Octopus 900 automatic perimeter, the background light 4asb, 60 detection points within 30° of the central area were selected for the VF examination. According to the Hodapp–Parrish-Anderson criteria, the 77 eyes of all 39 participants were divided into three groups based on the severity of glaucomatous VF loss: mild, moderate, and severe baseline VF defects [12]. All the assessments listed above were performed by the same experienced technician.

Statistical analysis was performed with SPSS software (SPSS for Windows, version 19.0; SPSS Inc., Chicago, IL). The simple linear regression analysis was used to identify trends within groups. *p* < 0.05 means the difference was statistically significant.

## 3. Results

As presented in Table 1, there were all the relevant demographic data the three groups at baseline. In our study, the surgery type of all patients who have been performed on surgery was trabeculectomy.

**Figure 1:**
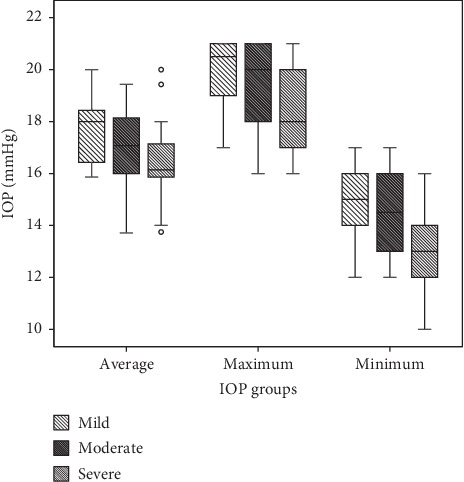
Box chart of the minimum, maximum, and average of IOP in three groups.

**Figure 2:**
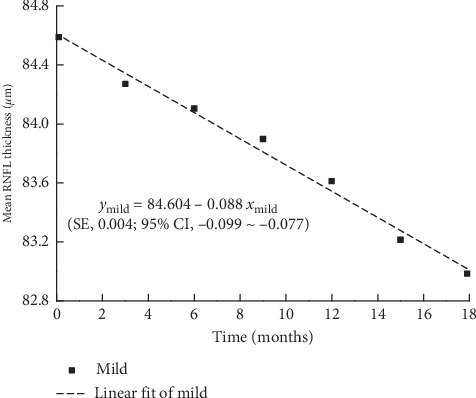
Scatter diagram of mean RNFL thickness in eyes with mild VF defect. SE: standard error of the slope; 95% CI: 95% confidence interval of the slope.

**Figure 3:**
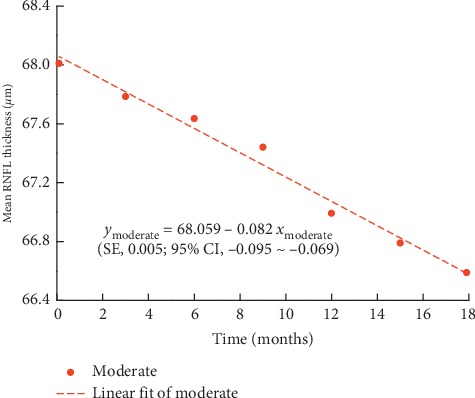
Scatter diagram of mean RNFL thickness in eyes with moderate VF defect. SE: standard error of the slope; 95% CI: 95% confidence interval of the slope.

**Figure 4:**
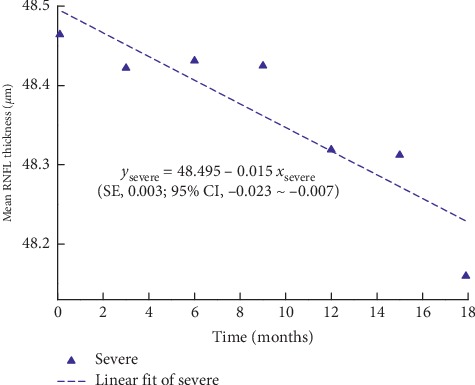
Scatter diagram of mean RNFL thickness in eyes with severe VF defect. SE: standard error of the slope; 95% CI: 95% confidence interval of the slope.

**Figure 5:**
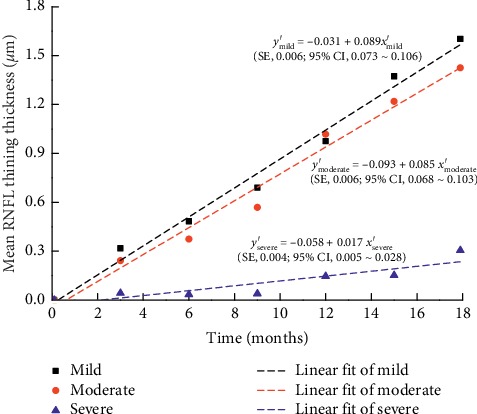
Scatter diagram of the mean RNFL thinning thickness in eyes with different degrees of VF defects. SE: standard error of the slopes; 95% CI: 95% confidence interval of the slopes.

The maximum, minimum, and average IOPs of the three groups after 18 months of follow-ups are presented in Figure 1. The mean RNFL thickness changes in patients with mild, moderate, and severe baseline VF defects are shown in Figures 2–4, respectively, and the mean thickness of the RNFL thinning for the three groups are presented in Figure 5. The RNFL thickness changes in the three groups of patients were basically consistent with a binary normal distribution.

As presented in Table 2, we used simple linear regression analysis to assess the correlation between the mean RNFL thickness and time in each group. The analysis of variance was used to prove the linear relationship between RNFL thickness and time. The *R*^2^ values were found to be 0.988, 0.982, and 0.814 for the mild, moderate, and severe defect groups, respectively. According to the changes in the RNFL thickness with time, we can obtain the following RNFL change formulas as a function of time for the three groups:

(1) $$\begin{array}{c} y_{\text{mild}}=84.604-0.088x_{\text{mild}},\\ y_{\text{moderate}}=68.059-0.082x_{\text{moderate}},\\ y_{\text{severe}}=48.495-0.015x_{\text{severe}}. \end{array}$$

In the above formulas, *y* represents the mean RNFL thickness, *x* represents the time since baseline, and −0.088, −0.082 and −0.015 represent the slopes for the mild, moderate, and severe groups, respectively. The standard errors of the slopes for the mild, moderate, and severe groups are 0.004, 0.005, and 0.003, and the confidence intervals of the slopes for them are −0.099∼−0.077, −0.095∼−0.069, and −0.023∼−0.007, respectively.

Table 2 shows the different thickness of the RNFL changes in patients with different baseline VF defects compared with baseline. Simple linear regression analysis was used to determine the correlation between RNFL thinning thickness and time in each group. The analysis of variance was used to prove the linear relationship between RNFL thinning thickness and time. The *R*^2^ values were found to be 0.982, 0.978, and 0.805 for the mild, moderate, and severe defect groups, respectively. According to the changes in RNFL thinning with time, we can obtain the following RNFL thinning formulas as a function of time for the three groups:

(2) $$\begin{array}{c} y_{\text{mild}}^{\prime }=-0.031+0.089x_{\text{mild}}^{\prime },\\ y_{\text{moderate}}^{\prime }=-0.093+0.085x_{\text{moderate}}^{\prime },\\ y_{\text{severe}}^{\prime }=-0.058+0.017x_{\text{severe}}^{\prime }. \end{array}$$

In the above formulas, *y*′ represents the mean thickness of RNFL thinning, *x*′ represents the time since baseline, and 0.089, 0.085, and 0.017 represent the slopes for the mild, moderate, and severe groups, respectively. The standard errors of the slopes for the mild, moderate, and severe groups are 0.006, 0.006, and 0.004, and the confidence intervals of the slopes for them are 0.073∼0.106, 0.068∼0.103, and 0.005∼0.028, respectively.

All the results shown above indicate that RNFL thinning occurs at a nearly linear rate, and the RNFL thinning rate is the fastest in individuals with mild baseline VF defects, followed by those with moderate defects. In individuals with severe VF defects, the linear change in RNFL thickness does not reflect the progression of the disease.

## 4. Discussion

Our results suggest that the rate of RNFL thinning is approximately linear. Harwerth and investigators found that RNFL thickness changes linearly as a function of the number of axons over the dynamic range of measurement [13]. Because the attenuation of the RNFL thickness is linear, we can obtain different slopes in RNFL changes when the disease is progressing and not progressing. We can determine whether the disease is progressing by comparing the actual slope of the RNFL changes with the slopes of progression or no progression.

Previous researchers only studied differences in RNFL thickness between people with and without POAG or the overall linear changes in the RNFL thickness or simply used RNFL thickness in combination with other indicators to diagnose and follow up patients, but they did not systematically study the changes in RNFL over time [13–15]. The slope of the RNFL changes differed across POAG patients with different severities of VF defects, and this result was mainly caused by the different structural characteristics of the eye in individuals with different VF defects. In our study, we performed regular OCT measurements for RNFL thinning to diagnose and follow up POAG patients at different stages to determine whether the RNFL thickness differs and how it differs in individuals with different degrees of VF defects. In our study, we found that the rate of RNFL thinning was the fastest in the early stage of POAG and the second fastest in the middle stage, while the thinning of the RNFL should not be used as an indicator of the progression of POAG in patients in the advanced stage. The following results from previous studies can explain our conclusion. In the early stage of the disease, the assessment of glaucoma progression is usually performed by detecting changes over time in structural and functional measurements [16]. Using standard structural testing to monitor advanced POAG is extremely difficult because standard structural measures have a limited dynamic range that becomes narrower with advanced POAG [1, 17–19]. In addition, structural assessments by imaging instruments seem to be relatively sensitive to changes in the early stage of the disease, whereas standard automated perimetry seems to perform relatively better with moderate and advanced stages of damage [15, 20]. According to the study above, RNFL changes are more pronounced in the early period than in the moderate and advanced periods, and RNFL changes are the least pronounced in advanced patients. Some investigators thought that an essential relationship exists between MD measured by perimetry and the thickness of the RNFL measured by OCT [21, 22]. Large changes in the RNFL thickness in eyes with early damage corresponded to small changes in the MD; however, for eyes with an advanced stage of the disease, large changes in the MD only corresponded to small changes or no changes in the average RNFL thickness [23]. This result also indicates that, for the same degree of VF progression, larger decreases in RNFL thickness occur in cases with mild damage than in those with severe damage. In addition, some investigators found that the rate of progression of VF defects is linear; mild defects progress the fastest, moderate defects progress the second fastest, and severe defects progress the slowest [9]. Therefore, RNFL thinning in the early stage of POAG is faster than that in the middle stage, while in the late stage, RNFL thinning is not obvious and should not be used as an indicator of disease progression.

Many POAG patients suffer from severe VF damage or even blindness during treatment, which is related to excessive dependence on the IOP to determine the adequacy of treatment. Differentiation of structural and functional changes in glaucoma plays an important role in the early diagnosis, treatment, and monitoring of the disease course [9]. Our study has shown that, in patients with POAG, disease progression is more likely to be detected by the measurement of structural changes in the early stage of the disease because the RNFL changes occur at the fastest rate in this stage, and structural assessments performed with imaging instruments are relatively sensitive to changes in the early stage. Therefore, for patients with mild baseline VF defects, especially those with VF defects in the early stage and who may have been in the early stage for a long time, strict intraocular pressure control and regular structural examinations, such as examinations of the thickness of the RNFL, are needed in this stage to track the disease progression over time. For patients with moderate VF defects, RNFL thinning tends to slow. Considering the difference in progression rates between the early and moderate groups is tiny, we think that RNFL inspection is still significant for the follow-up of POAG in the moderate stage. For patients with severe VF defects, the progression of the disease becomes harmful and can even cause blindness. However, the thinning of the RNFL should not be used to evaluate the progression of the disease in this stage. Thus, with strict and regular VF examinations, we should carry out more accurate examinations that do not depend excessively on the segmentation of the retina layer by layer, such as 3D stereoscopic nerve head (ONH) examinations [24].

In addition, regarding the impact of the age and surgery on the progress of the disease, we have the following explanations: Chauhan and other researchers found that the progression of the disease was related to aging. In their study, the median age of the subjects was 65.2 years [25], but the median age of our subjects tended to be younger and there exists little difference of age in each group; therefore, we did not take age-related effects into account. IOP level is related to the surgery, especially considering the 24-hour IOP fluctuation. But Yuan found that some areas with high tolerance to high IOP are likely to recover nerve function after surgery, but the disease may deteriorate as the follow-up period prolongs [26]. All postoperative subjects in our study were selected more than 3 months after surgery, and the type of surgery they performed is the same as trabeculectomy. Therefore, the effect of short-term IOP reduction on RNFL and the influence of the type of surgery are excluded.

The limitation of our study is that the follow-up and RNFL thickness analyses only focused on the trends in the average thickness of the RNFL in individuals with different VF defect stages, while the changes in the trends in the RNFL thickness in different parts of the eye were not specifically analyzed. In the future study, we will further explore the RNFL changes in different parts and combine the VF progression data and RNFL thickness changes to analyze the proportions of patients progressed in different stages of the disease. It is true that the old age has a certain effect on the disease progression. We will conduct a further study on the trends in the RNFL thickness changes to older cohorts in subsequent work.

In conclusion, our study showed that POAG patients can be followed up by structural examinations, such as RNFL thickness examinations in the mild stage, because RNFL thinning occurs at the fastest rate in this stage. In the moderate stage, RNFL inspection is still significant for the reason that the difference in progression rates between the mild and moderate groups is tiny. The RNFL thickness is not applicable for monitoring the progression of the advanced stage. Therefore, follow-ups should include VF measurements and other measurements that do not depend on layer-by-layer retinal segmentation.

## Figures and Tables

**Table 1:** Baseline characteristics of patients.

Group	Mild	Moderate	Severe
Example	21	18	38
Age (years)	39.9 ± 9.1	37.9 ± 8.2	43.5 ± 10.3
Gender (female/male)	4/17	5/13	13/25
Surgery (Y/N)	6/15	10/8	23/15
Mean IOP (mmHg)	17 ± 1.67	17.17 ± 1.86	16.36 ± 1.72
Mean MD (dB)	3.62 ± 1.41	8.59 ± 1.79	18.16 ± 3.57
Mean RNFL (*μ*m)	84.59 ± 18.35	68.01 ± 9.83	48.46 ± 9.14

**Table 2:** Thickness of mean RNFL and mean RNFL thinning compared with baseline $\left(\bar{\mu }m\right)$.

	Time	Mild (3.62 ± 1.41 dB)	Moderate (8.59 ± 1.79 dB)	Severe (18.16 ± 3.57 dB)
A	Baseline	84.5871	68.0094	48.4642
	At 3 months	84.2700	67.7853	48.4218
	At 6 months	84.1038	67.6359	48.4311
	At 9 months	83.8976	67.4412	48.4247
	At 12 months	83.6119	66.9924	48.3190
	At 15 months	83.2138	66.7906	48.3121
	At 18 months	82.9848	66.5888	48.1595
	*R*_*A*value_^2^	0.988	0.982	0.814
	*F*_*A*_(*P*)	418.279 (*p* < 0.05)	276.064 (*p* < 0.05)	21.884 (*p* < 0.05)

B	At 3 months	0.3171	0.2241	0.0424
	At 6 months	0.4833	0.3735	0.0331
	At 9 months	0.6895	0.5682	0.0395
	At 12 months	0.9752	1.0170	0.1452
	At 15 months	1.3733	1.2188	0.1521
	At 18 months	1.6023	1.4206	0.3047
	*R*_*B*value_^2^	0.982	0.978	0.805
	*F*_*B*_(*P*)	219.026 (*p* < 0.05)	175.948 (*p* < 0.05)	16.558 (*p* < 0.05)

A: mean RNFL thickness for each group; *R*_*A*value_^2^: use simple linear regression analysis to get the coefficient determination of each group; *F*_*A*_: use analysis of variance to prove the linear relationship between RNFL thickness and time. B: mean RNFL thinning thickness compared with baseline for each group; *R*_*B*value_^2^: use simple linear regression analysis to get the coefficient determination of each group; *F*_*B*_: use analysis of variance to prove the linear relationship between RNFL thinning thickness and time.

## Data Availability

The data supporting the results of the current article are available from the corresponding author upon request.

## References

[1] Hood D. C., Kardon R. H. (2007). A framework for comparing structural and functional measures of glaucomatous damage. *Progress in Retinal and Eye Research*.

[2] Varma R., Ying-Lai M., Francis B. A. (2004). Prevalence of open-angle glaucoma and ocular hypertension in Latinos. *Ophthalmology*.

[3] King A. J., Stead R. E., Rotchford A. P. (2011). Treating patients presenting with advanced glaucoma--should we reconsider current practice?. *British Journal of Ophthalmology*.

[4] Leighton P., Lonsdale A. J., Tildsley J., King A. J. (2012). The willingness of patients presenting with advanced glaucoma to participate in a trial comparing primary medical vs primary surgical treatment. *Eye*.

[5] Wall M., Kutzko K. E., Chauhan B. C. (1997). Variability in patients with glaucomatous visual field damage is reduced using size V stimuli. *Investigative Ophthalmology & Visual Science*.

[6] Keltner J. L., Johnson C. A., Levine A. (2005). Normal visual field test results following glaucomatous visual field end points in the ocular hypertension treatment study. *Archives of Ophthalmology*.

[7] Weinreb R. N., Friedman S., Fechtner D. (2004). Risk assessment in the management of patients with ocular hypertension. *American Journal of Ophthalmology*.

[8] Leung C. K. S., Yu M., Weinreb R. N. (2010). Retinal nerve fiber layer imaging with spectral-domain optical coherence tomography: patterns of retinal nerve fiber layer progression. *Ophthalmology*.

[9] Gustavo D. M., Jeffrey M. L., Leonard A. L. (2017). Detection and measurement of clinically meaningful visual field progression in clinical trials for glaucoma. *Progress in Retinal & Eye Research*.

[10] Bowd C., Zangwill L. M., Berry C. C. (2001). Detecting early glaucoma by assessment of retinal nerve fiber layer thickness and visual function. *Investigative Opthalmology & Visual Science*.

[11] Meiyu L. (2004). *Glaucoma*.

[12] Hodapp E., Parrish R. K., Anderson D. R. (1993). *Clinical Decisions in Glaucoma*.

[13] Harwerth R. S., Carter-Dawson L., Smith E. L., Crawford M. L. J. (2005). Scaling the structure−function relationship for clinical perimetry. *Acta Ophthalmologica Scandinavica*.

[14] Harwerth R. S., Wheat J. L., Fredette M. J., Anderson D. R. (2010). Linking structure and function in glaucoma. *Progress in Retinal and Eye Research*.

[15] Medeiros F. A., Zangwill L. M., Anderson D. R. (2012). Estimating the rate of retinal ganglion cell loss in glaucoma. *American Journal of Ophthalmology*.

[16] Sung K. R., Sun J. H., Na J. H., Lee J. Y., Lee Y. (2012). Progression detection capability of macular thickness in advanced glaucomatous eyes. *Ophthalmology*.

[17] Mwanza J.-C., Budenz D. L., Warren J. L. (2015). Retinal nerve fibre layer thickness floor and corresponding functional loss in glaucoma. *British Journal of Ophthalmology*.

[18] Mwanza J.-C., Kim H. Y., Budenz D. L. (2015). Residual and dynamic range of retinal nerve fiber layer thickness in glaucoma: comparison of three OCT platforms. *Investigative Opthalmology & Visual Science*.

[19] Hood D. C., Raza A. S., de Moraes C. G. V., Liebmann J. M., Ritch R. (2013). Glaucomatous damage of the macula. *Progress in Retinal and Eye Research*.

[20] Harwerth R. S., Vilupuru A. S., Rangaswamy N. V., Smith E. L. (2007). The relationship between nerve fiber layer and perimetry measurements. *Investigative Opthalmology & Visual Science*.

[21] Hee M. R., Izatt J. A., Swanson E. A. (1995). Optical coherence tomography of the human retina. *Archives of Ophthalmology*.

[22] Weymouth A. (2014). Optical coherence tomography of ocular diseases 3rdEdition by schuman JS, puliafito CA, fujimoto JG and duker JS thorofare, New Jersey, USA: slack Inc, 2013 615 pages RRP $358.84. *Clinical and Experimental Optometry*.

[23] Medeiros F. A., Zangwill L. M., Bowd C., Mansouri K., Weinreb R. N. (2012). The structure and function relationship in glaucoma: implications for detection of progression and measurement of rates of change. *Investigative Opthalmology & Visual Science*.

[24] Akram B., Medeiros F. A., Christopher B. (2016). Structural change can Be detected in advanced-glaucoma eyes. *Investigative Opthalmology & Visual Science*.

[25] Chauhan B. C., Mikelberg F. S., Artes P. H. (2010). [Canadian glaucoma study]: 3. Impact of risk factors and intraocular pressure reduction on the rates of visual field change. *Archives of Ophthalmology*.

[26] Wang W. J., Yuan Y. S. (2009). Progress of the visual field after primary open-angle glaucoma trabeculectomy in middle and late stage. *International Journal of Ophthalmology*.

